# A novel small RNA CoaR regulates coenzyme A biosynthesis and tolerance of *Synechocystis* sp. PCC6803 to 1-butanol possibly via promoter-directed transcriptional silencing

**DOI:** 10.1186/s13068-017-0727-y

**Published:** 2017-02-20

**Authors:** Tao Sun, Guangsheng Pei, Jiangxin Wang, Lei Chen, Weiwen Zhang

**Affiliations:** 1 Laboratory of Synthetic Microbiology, School of Chemical Engineering and Technology, Tianjin University, Tianjin, 300072 People’s Republic of China; 2 Key Laboratory of Systems Bioengineering (Ministry of Education), Tianjin University, Tianjin, 300072 People’s Republic of China; 3 SynBio Research Platform, Collaborative Innovation Center of Chemical Science and Engineering, Tianjin, 300072 People’s Republic of China; 4 Shenzhen Engineering Lab for Marine Algal Biotechnology, College of Life Science, Shenzhen University, Shenzhen, 518060 People’s Republic of China; 5 Center for Biosafety Research and Strategy, Tianjin University, Tianjin, People’s Republic of China

**Keywords:** sRNA, *Synechocystis*, Tolerance, 1-Butanol, CoA biosynthesis, Transcriptional silencing

## Abstract

**Background:**

Microbial small RNAs (sRNAs) have been proposed as valuable regulatory elements for optimizing cellular metabolism for industrial purposes. However, little information is currently available on functional relevance of sRNAs to biofuels tolerance in cyanobacteria.

**Results:**

Here, we described the identification and functional characterization of a novel 124 nt sRNA Ncl1460 involved in tolerance to biofuel 1-butanol in *Synechocystis* sp. PCC 6803. The expression of Ncl1460 was verified by blotting assay and its length was determined through 3′ RACE. Further analysis showed that Ncl1460 was a negative regulator of *slr0847* (*coaD*) and *slr0848* operon responsible for coenzyme A (CoA) synthesis possibly *via* promoter-directed transcriptional silencing mechanisms which has been widely discovered in eukaryote; thus Ncl1460 was designated as CoaR (CoA Biosynthesis Regulatory sRNA). The possible interaction between CoaR and target genes was suggested by CoA quantification and green fluorescent protein assays. Finally, a quantitative proteomics analysis showed that CoaR regulated tolerance to 1-butanol possibly by down-regulating CoA biosynthesis, resulting in a decrease of fatty acid metabolism and energy metabolism.

**Conclusions:**

As the first reported sRNA involved CoA synthesis and 1-butanol tolerance in cyanobacteria, this study provides not only novel insights in regulating mechanisms of essential pathways in cyanobacteria, but also valuable target for biofuels tolerance and productivity modifications.

**Electronic supplementary material:**

The online version of this article (doi:10.1186/s13068-017-0727-y) contains supplementary material, which is available to authorized users.

## Background

Bacterial small RNAs (sRNAs) are functional non-coding RNAs with a typical length of 50–300 nt, among which *trans*-acting sRNAs (ncRNAs) transcribe from the intergenic regions and function at a distance to alter the expression of target RNAs, while *cis*-acting sRNAs (asRNAs) transcribe from the complementary strand of the known open reading frames (ORFs) and regulate translation or decay mRNA molecules through base paring [1]. Photosynthetic cyanobacteria have been proposed as “autotrophic cell factories” for biofuels production due to its ability to utilize CO_2_ and sunlight directly for growth [2]. In cyanobacteria *Synechocystis* sp. PCC 6803 (hereafter *Synechocystis*), though previous studies have approximately identified 300 putative sRNAs [3], their functional characterizations still significantly lag behind. So far only several sRNAs of *Synechocystis* have been functionally revealed, including an asRNA IsrR functioning as a negative regulator of the CP43 homolog IsiA, a negative regulator named as_flv4 involved in response to shifts in inorganic carbon supply, PsbA2R and PsbA3R acting as positive regulators to enhance the stability of target mRNAs as well as PsrR1 controlling photosynthetic functions [4]. Recently, NsiR4 was found involved in nitrogen assimilation control [5].

1-Butanol biosynthetic pathways have been introduced into various cyanobacterial systems and its production could reach 404 mg/L in *Synechococcus elongatus* PCC 7942 and 37 mg/L in *Synechocystis* [6, 7]. However, current productivity is still much lower than the native *Clostridium* or even synthetic *Escherichia coli* systems [8, 9], partially due to the high toxicity of 1-butanol to cyanobacteria [2]. Recently, Kaczmarzyk et al. [10] overexpressed an RNA polymerase sigma factor *sigB* in *Synechocystis* and successfully enhanced the tolerance to 1-butanol in a butanol-shock experiment. Our recent efforts on studying metabolic responses of *Synechocystis* to various biofuels have also led to the discovery of two response regulator genes (i.e., *slr1037* and *sll0039*) related to 1-butanol tolerance [11, 12], and several transcriptional regulators (i.e., *sll0794*, *sll1392*, *sll1712*, and *slr1860*) related to ethanol tolerance [13, 14]. These studies demonstrated the possibility of improving biofuel tolerance by engineering transcriptional regulation. Previous studies have shown the vital roles of sRNAs in responding to environmental perturbations among various microbes through transcriptional or post-transcriptional regulation [1]. In a recent study, Gaida et al. [15] showed that overexpression of three sRNAs, DsrA, RprA, and ArcZ, could increase acid tolerance of *E. coli* up to 8500-fold. Meanwhile, the transcriptomic analysis of cellular responses to ethanol production in *Synechocystis* identified several regulated functionally unknown sRNAs including *ncl1740*, *ncl1390*, and *ncl1600* [16], suggesting that sRNAs could be involved in the regulation of biofuels tolerance.

In this work, we employed a RNA-seq approach to identify sRNAs related with exogenous 1-butanol tolerance in *Synechocystis*, which led to discover a novel sRNA Ncl1460 responsive to 1-butanol. Further analysis demonstrated that Ncl1460 was a negative regulator of *coaD* involving coenzyme A (CoA) biosynthetic pathway, and was accordingly designated as CoaR (CoA Biosynthesis Regulatory sRNA). The interaction between CoaR and target genes was verified by CoA quantification and GFP assays. Finally, quantitative proteomics analysis revealed the decreased CoA content caused by *coaR* overexpressing could down regulate fatty acid metabolism and energy metabolism thus led to a decreased 1-butanol tolerance. As the first reported sRNA involved CoA synthesis and 1-butanol tolerance in cyanobacteria, this study provides not only novel insights in regulating mechanisms of essential pathways in cyanobacteria, but also valuable target for biofuels tolerance and productivity modifications.

## Results

### Growth of *Synechocystis* under 1-butanol stress

**Fig. 1:**
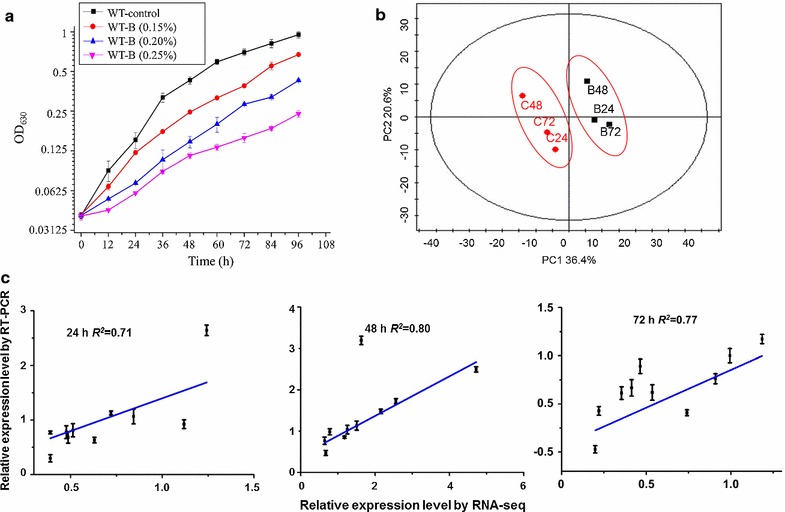
**a** Growth curves of WT in BG11 media with or without different concentrations (*v/v*) of 1-butanol. The *error bars* represent the calculated standard deviation of the measurements of three biological replicates. **b** PCA analysis of sRNome. C24, C48, C72 and B24, B48, B72 represent samples cultured at 24, 48, and 72 h in normal BG11 medium or BG11 medium supplemented with 1-butanol, respectively. **c** Validation of sRNome by qRT-PCR. The *error bars* represent the calculated standard deviation of the measurements of three biological replicates

Effects of 1-butanol on *Synechocystis* were performed using wild-type (WT) in BG-11 media supplemented without or with 1-butanol at a concentration of 0.15, 0.20 and 0.25% (*v/v*), respectively (Fig. 1a). Consistent with previous study [17], the results showed that 0.20% (*v/v*) 1-butanol caused about 50% growth inhibition at 48 h, and this concentration was selected for the transcriptomic analysis. WT cultivated with or without 1-butanol (0.20%, *v/v*) were collected at 24, 48, and 72 for small RNA-seq analysis, respectively, named accordingly as C24, C48, and C72 for control as well as B24, B48, and B72 for butanol-treated samples, respectively.

f

### sRNome analysis

After adapter trimming and data filtering, a total of 9,669,974, 13,383,938, 6,919,528, 9,938,132, 6,951,722, and 8,373,222 reads were obtained in six samples (i.e., C24, C48, C72 and B24, B48, B72, respectively). Then the sRNA reference libraries of *Synechocystis* (including approximately 1,071 known asRNAs and 320 known ncRNAs) described previously by Mitschke et al. [3] were used to identify the sRNAs in our study. Although the effective sequencing depth may vary in different samples, the obtained reads were able to match to approximately 1000 asRNAs and 290 ncRNAs (Additional file 2: Table S1), suggesting a good coverage.

Reads per kilobase per million mapped reads (RPKM) was employed to normalize the raw reads and a principal component analysis (PCA) was used to visualize the sRNomic expression profiles. The score plot showed that 1-butanol-treated samples (i.e., B24, B48 and B72) were visibly separated from the control samples (i.e., C24, C48, and C72) (Fig. 1b), suggesting that significant responses existed after 1-butanol treatment. Comparative expression analysis of the identified sRNAs showed that a total of 98, 168, 121 asRNAs and 42, 60, 43 ncRNAs were differentially expressed at 24, 48, and 72 h after 1-butanol treatment, respectively, using a criterion of the fold change >1.5 and *p* values ≤0.05 (Additional file 2: Table S2). To validate the reliability, 10 sRNAs were randomly selected for a quantitative real-time RT-PCR (qRT-PCR) analysis (Primers were listed in Additional file 2: Table S3.). Comparative qRT-PCR analysis between the butanol-treated and control samples showed a significant correlation with a correlation coefficient *R* ^2^ > 0.70 using the *Pearson* correlation analysis (Fig. 1c), suggesting the high reliability of the sRNomic analysis.

### sRNA Ncl1460 involved in 1-butanol stress response

**Fig. 2:**
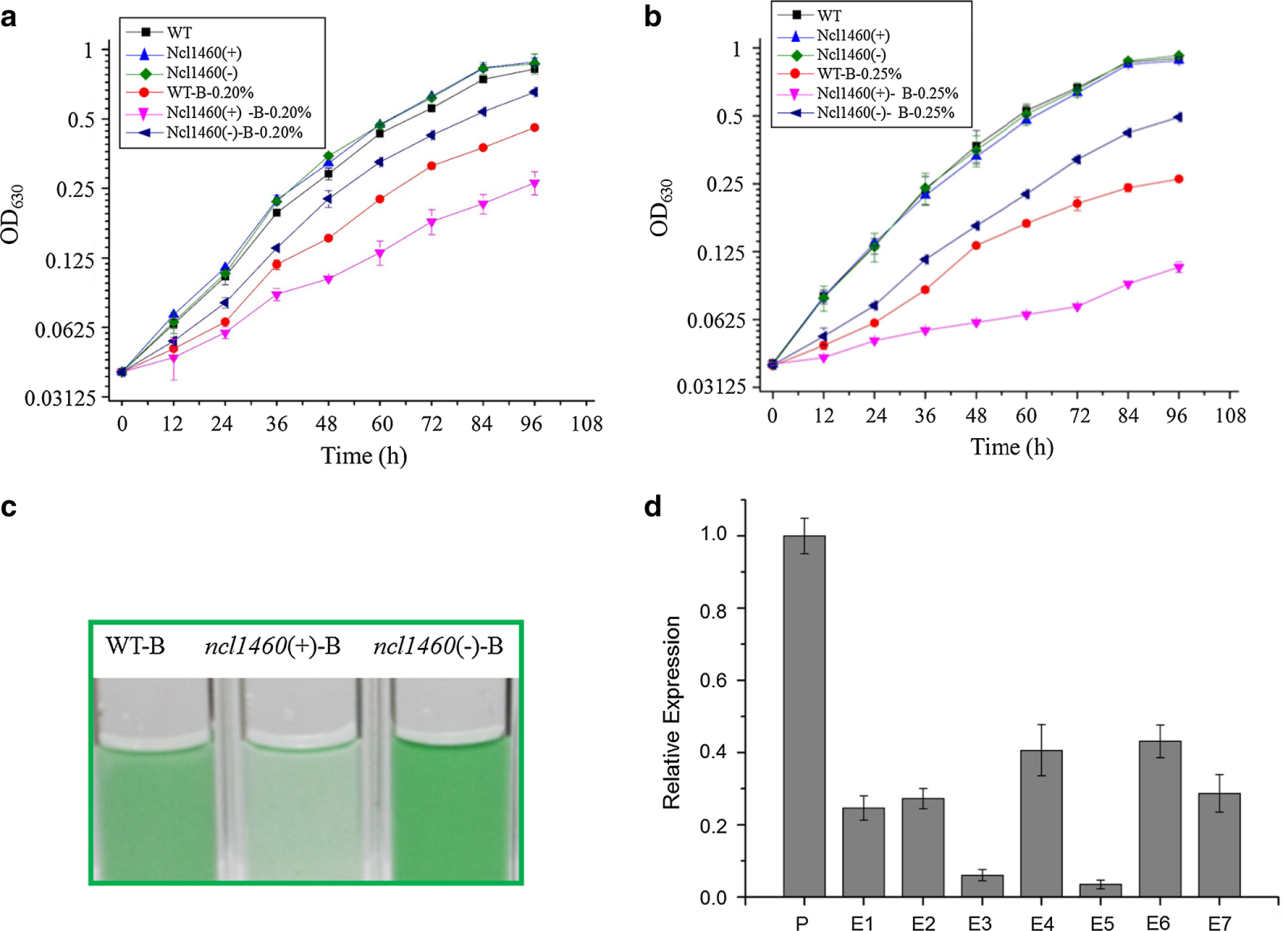
**a** Growth patterns of WT, Ncl1460(+), and Ncl1460(−) in BG11 media with or without 0.20% (*v/v*) 1-butanol. The *error bars* represent the calculated standard deviation of the measurements of three biological replicates. **b** Growth patterns of WT, Ncl1460(+), and Ncl1460(−) in normal BG11 media with or BG11 without 0.25% (*v/v*) 1-butanol. The *error bars* represent the calculated standard deviation of the measurements of three biological replicates. **c** Growth phenotypes of WT, Ncl1460(+), and Ncl1460(−) in BG11 medium with 0.25% (*v/v*) 1-butanol. **d** Relative expression levels of Ncl1460 in selected butanol-adapted strains to WT. E1, E2, E3, E4, E5, E6, and E7, respectively, represent the butanol-adapted strains that endure 0.20, 0.25, 0.30, 0.35, 0.40, 0.45, and 0.50% 1-butanol (*v/v*). The *error bars* represent the calculated standard deviation of the measurements of three biological replicates

A series of differentially regulated ncRNAs were selected for constructing overexpression [sense fragments of sRNA, expressed as sRNA(+)] and suppression strains [antisense fragments of sRNA, expressed as sRNA(−)] using a replicating vector pJA2 [10] and the phenotypic difference was examined under 0.20% (*v/v*) 1-butanol stress (Additional file 2: Table S4; Additional file 1: Fig. S1A) [18]. Although most of the mutants constructed showed no growth difference from WT, Ncl1460(+) (i.e., *Synechocystis* with overexpressed *ncl1460*) became more sensitive to 0.20% (*v/v*) 1-butanol than WT (Fig. 2a). This phenotype was even more significant under high 1-butanol concentration like 0.25% (*v/v*) (Fig. 2b, c). Interestingly, Ncl1460(−) (i.e., *Synechocystis* with suppressed *ncl1460*) became more tolerant to 1-butanol than WT (Fig. 2a, b). The overexpression and suppression of Ncl1460 in Ncl1460(+) and Ncl1460(−) were confirmed by reverse transcriptional PCR (RT-PCR) using WT as a control (Additional file 1: Fig. S3A), which clearly showed that the transcriptional level was increased in Ncl1460(+) but decreased in Ncl1460(−), respectively.

The above phenotype indicated that *ncl1460* may be a negative regulator during 1-butanol stress response. In the sRNomic data, the RKPM values for Ncl1460 were 14.52, 8.40, and 11.37 under control condition at 24, 48, and 72 h, respectively, while they respectively decreased to 5.65, 5.66, and 4.03 after 1-butanol treatment, indicating a remarkably decrease of the relative expression by 2.5-, 1.5-, and 2.9-fold (Additional file 2: Table S1). To verify whether Ncl1460(+) was sensitive specifically to 1-butanol, growth patterns were also determined under Cd^2+^ (4.6 μM), low pH (pH = 6.1) and NaCl (4.0% *w/v*) stress conditions. However, no differences were observed under Cd^2+^, NaCl, and low pH conditions, thus we focused on 1-butanol tolerance in this study.

### Northern blotting and 3′ RACE of Ncl1460

To verify the expression of Ncl1460, northern blotting assays was conducted using the specific probe of Ncl1460 and the results shown in Additional file 1: Fig. S3B clearly demonstrated its presence as a sRNA. In a previous study, the 5**′** end of Ncl1460 has been determined while its 3**′** end reminded unclear since the longest transcript among the several candidate transcripts for *ncl1460* through deep sequencing was simply selected in the previous study [19]; here, we determined the 3**′** end using 3**′** RACE (Additional file 1: Fig. S3C). After Sanger sequencing, the corrected length of Ncl1460 was confirmed as 124 nt, which was 55 nt shorter than previously reported (179 nt) [3].

### sRNA Ncl1460 up-regulated in butanol-adapted strains

In our previous study, Wang et al. [20] achieved a 150% increase in tolerance to 1-butanol in *Synechocystis* after a continuous 94 passages with gradually increased 1-butanol concentration from 0.20 to 0.50% (*v*/*v*). As *ncl1460* involve in 1-butanol tolerance, its expression may also be regulated during the evolutionary process. To prove the hypothesis, we applied a qRT-PCR analysis to measure the relative expression levels of *ncl1460* in seven evolutionary strains enduring different concentrations of 1-butanol. The results showed that relative expression level of *ncl1460* decreased clearly in all the evolutionary strains, and it was even lower in E3 and E5 which can endure 0.30 and 0.40% 1-butanol (*v/v*) than that in WT, respectively, (Fig. 2d), suggesting that besides coding genes, the transcriptional level of non-coding sRNAs could also change in laboratory-based adaptive strains.

### sRNA Ncl1460/CoaR as a negative regulator for coenzyme A biosynthesis

**Fig. 3:**
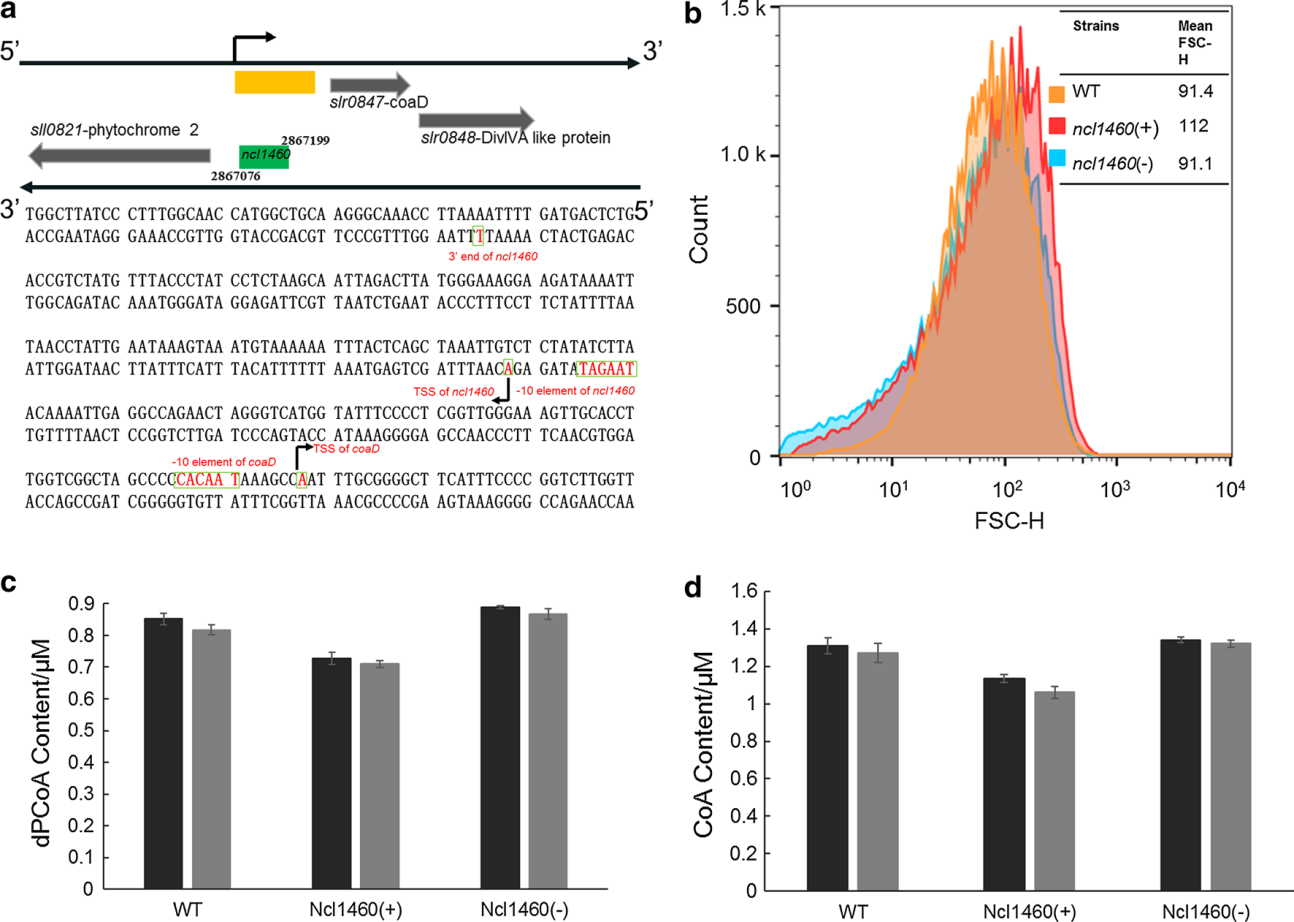
**a** Genetic location and detailed sequence information of Ncl1460 in the *Synechocystis* genome. **b** Cell size measurements of the WT, Ncl1460(+) and Ncl1460(−) strains. **c** Quantitation of dPCoA in the WT, Ncl1460(+) and Ncl1460(−) strains. **d** Quantitation of CoA in the WT, Ncl1460(+) and Ncl1460(−) strains. Samples were harvested at 48 h and the *error bars* represent the calculated standard deviation of the measurements of three biological replicates

*ncl1460* was located between genes encoding a phytochrome-like protein (Sll0821) and a phosphopantetheine adenylyltransferase (PPAT, Slr0847) (Fig. 3a). To identify potential target genes regulated by Ncl1460, the CopraRNA software developed by Wright et al. (2014) was employed for target prediction [21] and six cyanobacteria genomes (i.e., NC_000911, NC_008312, NC_009091, NC_009840, NC_011738, NC_007776) were taken as the sRNA homologs for inputs [22]. The result found one of the flanking genes *slr0847* ranked first in the candidates (Additional file 1: Fig. S4). In *Synechocystis*, PPAT encoded by *slr0847* was the only enzyme reported so far to catalyze the conversion from pantotheine 4′-phosphate to 3′-dephospho-CoA (dPCoA) in CoA biosynthesis [23]. In addition, *slr0847* was found to be organized into one possible operon with *slr0848* encoding a hypothetical protein which led to an enlarged cell size after deletion [24, 25]. As *ncl1460* located upstream the *slr0847* on the complementary strand, we hypothesized that Ncl1460 may regulate *slr0847* by directly base paring. Transcriptional starting site (TSS) of *slr0847* has been identified in previous study and Ncl1460 was on its upstream but not within the transcribed region of *slr0847* (Fig. 3a), excluding the possibility that Ncl1460 regulated *slr0847* through post-transcriptional mechanisms by base paring [19]. As the possible interaction region between *ncl1460* and upstream region of *slr0847* was clearly predicted by CopraRNA (Additional file 1: Fig. S5), suggesting other mechanism may exist. In eukaryotes like *Saccharomyces cerevisiae*, plants and even mammalian cells, promoter-directed transcriptional silencing mediated by siRNA, have been discovered in recent years [26]. Under this mechanism, sRNA located beyond the TSS of target genes and silenced targets through mediating the methylation of promoters [27]. To verify whether Ncl1460 could affect its potential targets *slr0847* and *slr0848*, we examined the intracellular contents of dPCoA, CoA as well as the cell size differences between WT, Ncl1460(+) and Ncl1460(−) under both normal BG11 or BG11 medium with 0.25% (*v/v*) 1-butanol. Using liquid chromatography–mass spectrometry (LC/MS), the intracellular contents of dPCoA and CoA in Ncl1460(+) strain were found decreased by 14.7 and 13.5% under normal medium as well as 13.1 and 15.5% under 1-butanol stress condition, respectively. Although it is not significant, their abundances especially the abundance of dPCoA were increased by 4 and 6% in Ncl1460(−) strain compared with WT respectively under normal medium or 1-butanol stress condition (Fig. 3c, d). Furthermore, flow cytometry analysis showed that the mean size of Ncl1460(+) was enlarged than that of WT while the size of Ncl1460(−) cells showed no significant difference (Fig. 3b), consistent with the previous report [25]. These results further indicated that Ncl1460 was a negative regulator of *slr0847* and *slr0848* operon which could affect the CoA biosynthesis and cell size; thus, the sRNA Ncl1460 was renamed as CoaR (CoA Biosynthesis Regulatory sRNA).

### GFP assays in *Synechocystis*

**Fig. 4:**
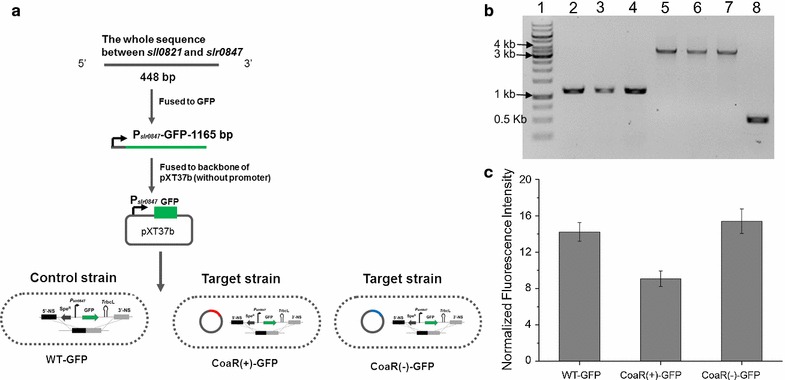
**a** Scheme of the GFP assays. **b** PCR validation of WT-GFP, CoaR(+)-GFP and CoaR(−)-GFP. *Lane1*: DNA ladder; *Lane 2*, *3*, and *4*: genomic DNA of WT-GFP, CoaR(+)-GFP and CoaR(−)-GFP as templates, respectively; primers P*slr0847*-F and GFP-R were applied (Additional file 2: Table S3) and the target size was 1165 bp; *Lane 5*, *6*, and *7*: genomic DNA of WT-GFP, CoaR(+)-GFP, CoaR(−)-GFP, and WT as templates, respectively; primers 0168-F and 0168-R were applied (Additional file 2: Table S3) and the target size was approximately 3600 bp for WT-GFP, CoaR(+)-GFP and CoaR(−)-GFP, 500 bp for WT; **c** GFP fluorescence in the WT-GFP, CoaR(+)-GFP, and CoaR(−)-GFP strains. The *error bars* represent the calculated standard deviation of the measurements of three biological replicates

To demonstrate the possible interaction between CoaR and the promoter of *slr0847*, the whole intergenic sequence between *sll0821* and *slr0847* (containing the promoter of *slr0847* named P_*slr0847*_) was amplified and fused to the GFP coding gene. The fused fragment was then ligated to backbone of pXT37b (without original promoter, Additional file 1: Fig. S1B) and introduced into WT, CoaR(+) and CoaR(−) strains (Fig. 4a). Full segregation of the transformants (i.e., WT-GFP, CoaR(+)-GFP, and CoaR(−)-GFP) were achieved by consecutively passaging and verified by PCR (Fig. 4b). Fluorescence of WT-GFP, CoaR(+)-GFP, and CoaR(−)-GFP was measured after cultivation for 48 h using a HITACHI F-2700 fluorescence spectrophotometer. The fluorescence intensity was normalized by optical density (OD_730_) of each sample and the basal fluorescence of WT. The results illustrated in Fig. 4c showed that the fluorescence intensity of CoaR(+)-GFP was decreased significantly than that of WT-GFP and CoaR(−)-GFP, demonstrating that the function of CoaR may functioned by silencing the promoter region of *slr0847*.

### Quantitative proteomics and lipid profiles reveals possible tolerance mechanisms

**Fig. 5:**
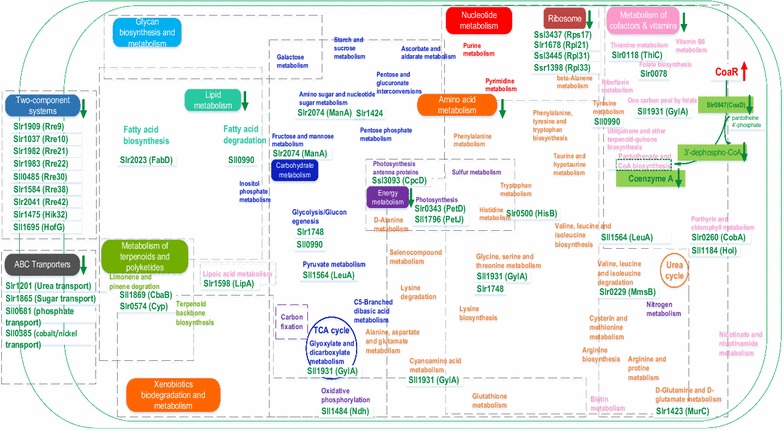
Proposed regulatory networks mediated by CoaR. The different metabolisms in *Synechocystis* were illustrated as *different colors* (e.g., *yellow* for amino acid metabolism and purple for energy metabolism). The down-regulated genes were listed as *green down arrows*

Finally, a quantitative proteomic analysis was utilized to identify the differential metabolic responses to 1-butanol between the WT and CoaR(+) strains. Samples of WT and CoaR(+) were harvested after cultivation with (0.20%, *v/v*) 1-butanol for 48 h, each with two biological replicates for proteomic analysis. After data filtering, the qualified spectra were matched to 2036 proteins (Additional file 1: Fig. S7). Comparative analysis showed that 159 proteins were down-regulated while 166 proteins up-regulated in the CoaR(+) strain using a 1.2-fold change as cut-off (*p* value <0.05) compared to WT (Additional file 2: Table S5). Proteomic data showed that the relative abundance of Slr0847 was decreased by 20% in the CoaR(+) strain, in good agreement with the 15.5% decrease of the CoA content in the same cells. CoA is an essential cofactor for numerous metabolic and energy-yielding reactions, also notable for its role in fatty acids metabolism and energy metabolism [28]. Consistently, the proteomic results showed that a wide range of metabolic changes including fatty acids metabolism, energy metabolism, amino acids metabolism, signal conduction, ABC transporters, ribosome metabolism, and cofactor biosynthesis were differentially regulated in the CoaR(+) strain, probably directly or indirectly due to the decreased cellular content of CoA in CoaR(+) under 1-butanol stress (Fig. 5).

The changes of intercellular CoA abundance could directly affect fatty acids biosynthesis and amino acids metabolism, and these metabolisms have been found related to tolerance to biofuels previously [29]. Malonyl-CoA-acyl carrier protein transacylase (FabD, Slr2023) was found down-regulated in CoaR(+) (Fig. 5). FabD was a key enzyme in the fatty-acid biosynthesis pathway, catalyzing the transfer of a malonyl moiety from malonyl-CoA to holo acyl carrier protein (ACP) [30]. Early studies have shown that increased long-chain fatty acids could enhance biofuel tolerance like ethanol in *Thermoanaerobacter ethanolicus* [31]. In addition, we also found that serine hydroxymethyltransferase (GylA) and phosphoglycerate mutase (Slr1748) involving glycine, serine, and threonine metabolism were down-regulated in the CoaR(+) strain and they have been found up-regulated in *Synechocystis* upon 1-butanol stress previously [17]. To determine whether the fatty acid biosynthesis was affected, we measured the total lipid profiles of WT, CoaR(+), and CoaR(−). Total lipids were extracted from approximately 5 mg dry cells of WT, CoaR(+), and CoaR(−), each with 3 biological replicates, then subjected to gas chromatography (GC) analysis following the protocol of Liu et al. [32]. The peak areas of each fatty acid were normalized by the dry weight of each sample. The results showed that the total lipid per milligram dry weight in the CoaR(+) strain was decreased by approximately 7.0%, compared with WT (Table 1). In addition, components of C14:1n9, C16:0, C18:1n9, C18:2n6, and C18:3n6 were found with a slight decrease in the CoaR(+) strain compared to WT. Interestingly, although the total lipid content in the CoaR(−) strain was approximately the same as that in WT, C14:1n9 and C18:3n6 decreased in CoaR(+) were increased in CoaR(−), which could be resulted from the repression of *coaR* in CoaR(−), consistent with the previous study in *Haematococcus pluvialis* finding the relative abundance of C18:3n6 was increased in cells under nitrogen depletion and low-temperature conditions [33].

**Table 1:** Lipid profiles of WT, CoaR(+), and CoaR(−)

	WT	CoaR(+)	CoaR(−)	CoaR(+)/WT	CoaR(−)/WT
C14:1n9	0.85 ± 0.027	0.82 ± 0.056	0.87 ± 0.038	0.96 ± 0.073	1.02 ± 0.055
C16:0	26.77 ± 0.087	23.99 ± 0.99	25.92 ± 0.13	0.9 ± 0.037	0.97 ± 0.006
C16:1n7	3.69 ± 0.031	3.67 ± 0.20	3.68 ± 0.24	0.99 ± 0.055	1 ± 0.066
C16:2n4	0.35 ± 0.004	0.37 ± 0.025	0.43 ± 0.042	1.05 ± 0.073	1.23 ± 0.12
C16:4n4	0.53 ± 0.024	0.55 ± 0.017	0.52 ± 0.033	1.03 ± 0.056	0.97 ± 0.076
C18:1n9	1.37 ± 0.058	1.32 ± 0.08	1.46 ± 0.056	0.96 ± 0.071	1.06 ± 0.061
C18:1n7	0.28 ± 0.029	0.37 ± 0.018	0.45 ± 0.003	1.33 ± 0.15	1.62 ± 0.17
C18:2n6	7.88 ± 0.69	7.39 ± 0.48	7.75 ± 0.52	0.94 ± 0.10	0.98 ± 0.11
C18:3n6	7.26 ± 0.21	7.00 ± 0.52	7.56 ± 0.44	0.96 ± 0.077	1.04 ± 0.068
Total area	48.97 ± 1.23	45.47 ± 1.26	48.61 ± 1.16	0.93 ± 0.035	0.99 ± 0.034

Content of each type of fatty acids was shown as the normalized peak area (area/mg) by GC. The first three columns were the mean peak area and the standard deviation of each fatty acid as well as the total area from three replicates. The last two columns represent the relative content of each fatty acid as well as the total lipid in CoaR(+) or CoaR(−) to that in WT

The energy metabolism is also directly related with CoA [28]. Four proteins (i.e., Slr0343, Sll1796, Ssl3093, and Sll1484) related with energy metabolism and photosynthesis process were down-regulated in the butanol sensitive CoaR(+) strain. Slr0343 (PetD) and Sll1796 (PetJ) were important parts of cytochromes and played vital roles in electron transfer [34]. Ssl3093 (CpcD) is a phycobilisome small rod linker polypeptide and involved in light harvesting. Sll1484 (Ndh) is a type-2 NADH dehydrogenase serving as redox-active electron transport intermediates [35]. Up-regulation of Slr0343 was reported previously under thermo-stress condition and its increased abundance allowed for rapid electron transfer from PSII to PSI [35]. In addition, Sll1796 was found significantly up-regulated in our previous transcriptomic and proteomic studies of *Synechocystis* under 1-butaonl stress [17, 36], and Ssl3093 was found significantly up-regulated in *Synechocystis* upon ethanol stress [37].

Besides the pathways directly related with CoA, five ABC transporters and nine two-component signal transduction system (TCS) proteins were found down-regulated in the butanol sensitive CoaR(+) strain (Fig. 5). ABC transport proteins have been suggested as an important mechanism against biofuel toxicity [38]. Two-component systems have been proved involving regulation of multiple stress conditions [39]. Chen et al. [11] recently found that the deletion of *slr1037* gene encoding a response regulator in *Synechocystis* led to a decreased tolerance to 1-butanol; consistently, our proteomic analysis also showed that Slr1037 was down-regulated in CoaR(+). Nevertheless, it is still unclear of the functional relationship between CoaR and Slr1037.

## Discussion

**Fig. 6:**
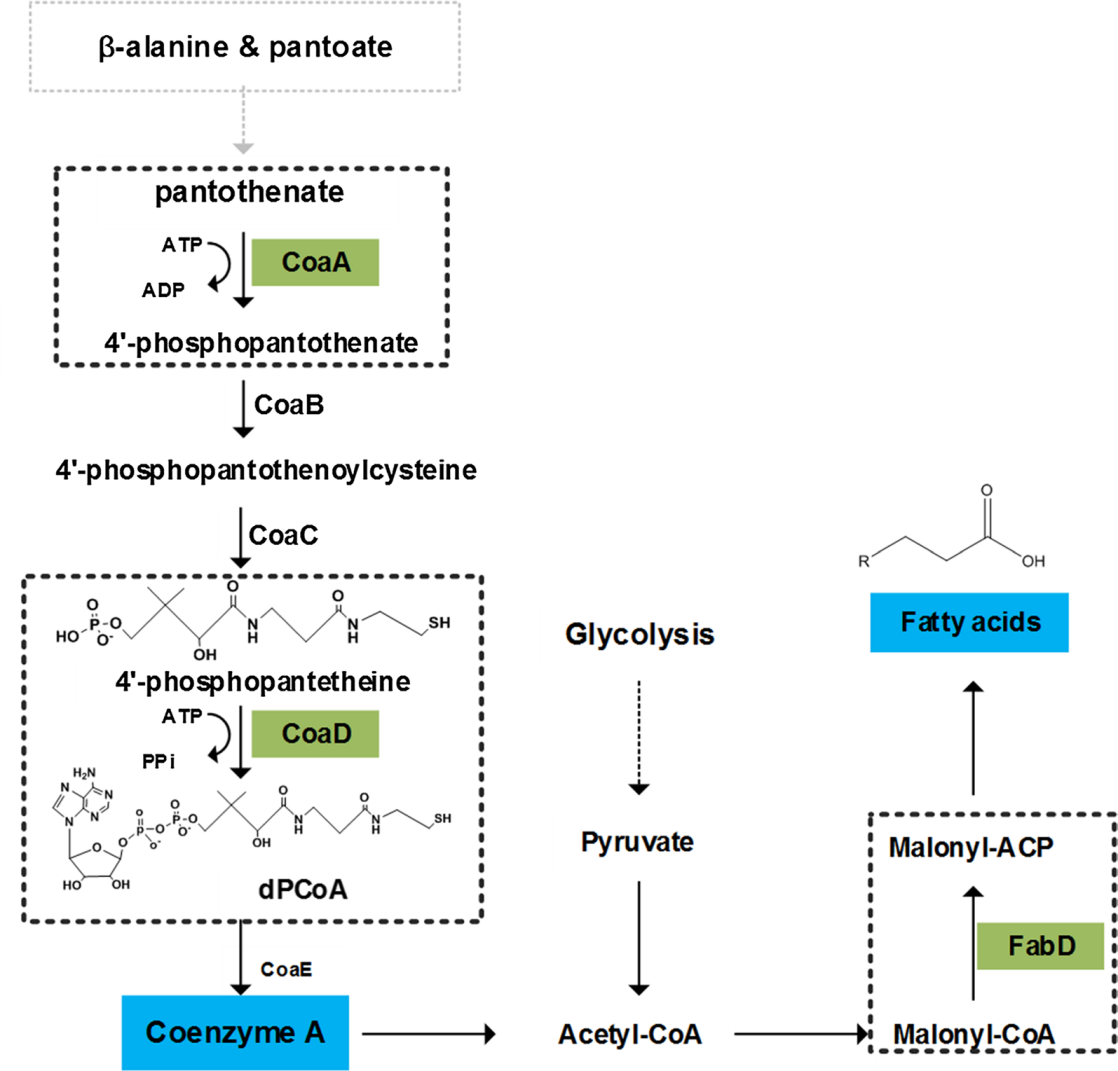
Biosynthetic pathways of CoA and fatty acids. The *gray-dashed boxes* and *green rectangle* represented the key regulated steps and enzymes during CoA and fatty acids biosynthesis, respectively

It is estimated that CoA is utilized by 9% of known enzymes and involved in over 100 reactions in cells [40]. In bacteria, CoA biosynthesis includes five enzymatic steps starting from pantothenate (Fig. 6) [41]. Given the fact that CoA is an essential cofactor in all living organisms [42], its regulation has attracted significant attentions. Early studies found that pantothenate kinase (CoaA) catalyzing the first step as well as phosphopantetheine adenylyltransferase (CoaD) in the fourth step were two key regulatory points in CoA biosynthesis process [43] (Fig. 6). Homologues of the *coaD* gene have been identified in a wide range of bacterial species, sharing a similar size (140–169 residues) and a high degree of sequence homology (32–52% identical) to the *E. coli* counterpart [44]. In addition, an early study showed that expression of *coaD* was regulated through a feedback of end-product CoA in *Pseudomonas aeruginosa* [45]. For *Synechocystis*, although no experimental information is available, *slr0847* is annotated as *coaD* involved in catalyzing the penultimate step of the CoA synthesis [23]. Besides, the identity between Slr0847 and *E. coli* CoaD was 48% as revealed by *Blast*-*p* (http://blast.ncbi.nlm.nih.gov/Blast.cgi?PAGE=Proteins). The function of *slr0847* homologue has been investigated in *Arabidopsis thaliana* through suppression, complementation, and overexpression [41], and the results showed that with a 90% reduction of CoaD in *A. thaliana*, root growth, seed production and the fatty acid content were all negatively affected while the complementation of this gene recovered these effects, suggesting the essential roles of *coaD* in growth and fatty acid biosynthesis in plants [41]. So far no sRNA has been reported for regulatory roles in CoA biosynthesis in any species to our knowledge. Thus, CoaR could be the first sRNA with direct roles in regulating CoA biosynthesis in model cyanobacterium *Synechocystis*.

Significant increase of tolerance against salt and osmotic stresses has previously been observed when overexpressing *coaD* in *A. thaliana*, suggesting *coaD* and CoA biosynthesis could be related with responding to environmental stress [41]. Consistently, the decreased expression of *coaD* by overexpressing *coaR* led to a decreased tolerance to 1-butanol while suppressing *coaR* resulted in higher tolerance to 1-butanol. Although the dPCoA and CoA changes were not significant (which may be due to the low abundances of intracellular dPCoA and CoA), the dPCoA and CoA contents in CoaR(−) had a slight increase, when compared with WT (Fig. 3c, d). Roles of sRNAs in biofuel stresses have been reported in other species. Venkataramanan et al. [46] recently discovered 159 sRNAs in responding to the native but toxic metabolites 1-butanol and butyrate in *C. acetobutylicum*. In addition, Cho et al. [47] validated 3 sRNAs (i.e., Zms2, Zms6, and Zms18) with differential expression in *Z. mobilis* under 5% ethanol stress. In addition, Aguilar et al. [48] found that sRNAs *omrA* and *omrB* regulating protein composition of the outer membrane were site-mutated after an adaptive laboratory evolution process of *E. coli* in glucose for 120 h. Here we found the transcriptional level of *coaR* also changed in 1-butanol adaptive evolution process, suggesting the important roles of sRNAs in biofuels response. Unlike overexpression of genes, regulation mediated by sRNA hardly imposes any metabolic burden on cells, and represents a more effective strategy for strains modification [15]. Here we identified the sRNA CoaR related to biofuel tolerance through regulating CoA biosynthesis pathways, constituting a valuable basis for further sRNA engineering in cyanobacteria in the future.

Our studies via quantitative proteomics showed that CoA was involved in the 1-butanol stress response mainly through affecting the fatty acid biosynthesis and energy metabolism. In *Bacillus subtilis*, fatty acid biosynthetic genes including the *fab* initiation genes *fabHB* and *fabD* as well as the elongation genes *fabF*, *fabG*, and *fabI* were found significantly up-regulated after sorbic acid exposure [49]. Consistently, our proteomics analysis also revealed the reduction of FadD in the CoaR(+) strain after 1-butanol exposure, and the down-regulation of FadD caused by decreased CoA may affect the membrane remodeling thus contributed to the low 1-butanol tolerance. In a study of *E. coli* in responding to heat shock stress, it was shown that intracellular ATP levels could transiently increase to meet the higher consumption for protein- and DNA-repair mechanisms [50]; besides, decreased expression of amino acyl-tRNA synthetases might promote the release of amino acids that feed energy-providing pathways [51]. Consistently, we found in this study, the decreased abundance of energy metabolism related proteins (i.e., Slr0343, Sll1796, Ssl3093 and Sll1484) and ribosome metabolism related proteins (i.e., Rps17, Rpl21, Rpl31 and Rpl33), could also lead to a sensitive phenotype of CoaR(+) to 1-butanol.

Finally, although we proposed a possible mechanism for CoaR based on our study, we cannot exclude other possible mechanisms as the direct interaction mechanism between *ncl1460* and *slr0847* has not been verified experimentally. Nevertheless, this mode of mechanism has been well studied and demonstrated in eukaryotes like yeast, plants and mammalian cells [26]. In addition, previous studies found that almost all relevant regulatory genes can only affect tolerance at a small degree individually in *Synechocystis* suggesting that the tolerance may be regulated by multiple regulatory genes simultaneously [11–14], which is also consistent with the excellent results of enhancing acid tolerance significantly by overexpressing three sRNAs (DsrA, RprA, and ArcZ) in *E. coli* [15]. We believe with more of the tolerance-relevant sRNAs identified in the future, together with this finding, a better understanding of the tolerance regulation in *Synechocystis* will be eventually achieved.

## Conclusions

This study found a novel sRNA CoaR regulating CoA synthesis as well as the tolerance of *Synechocystis* to 1-butanol. Overall, the study provided novel insight to the regulation of CoA biosynthesis in cyanobacteria, and also indicated that sRNAs could be useful targets in improving biofuel tolerance.

## Methods

### Bacterial growth conditions

For *Synechocystis*, WT and mutants were grown on BG11 agar plate or in medium (pH 7.5) under a light intensity of approximately 50 μmol photons m^−2^ s^−1^ in an illuminating incubator or shaking incubator at 130 rpm at 30 °C [17]. Medium for different mutants in this study was supplemented with appropriate antibiotic(s) to maintain plasmids with sRNAs or genes fused to genome (i.e., 10 μg/mL kanamycin, 10 μg/mL spectinomycin or a combination). For *E. coli*, DH 5α were grown on LB agar plate or in LB medium with appropriate antibiotic(s) to maintain plasmids (i.e., 100 μg/mL ampicillin) at 37 °C using incubator or shaking incubator at 200 rpm, respectively.

### SRNome sequencing and data analysis

Small RNA-seq transcriptome sequencing was carried out as described previously [17]: (1) Samples composition: WT grew under BG11 medium with or without 0.20% (*v*/*v*) 1-butanol were collected for the transcriptome analysis at 24, 48, and 72 h, respectively; (2) RNA preparation and cDNA synthesis: approximately 10 mg of cell pellets was frozen by liquid nitrogen immediately after centrifugation. Total RNA extraction of 6 samples was achieved through a miRNeasy Mini Kit (Qiagen, Valencia, CA) following the protocols, and 500 ng total RNA was subjected to cDNA synthesis using a NuGEN Ovation^®^ Prokaryotic RNA-seq System (NuGEN, San Carlos, CA); (3) RNA sequencing: RNA 2 × 100 bp paired-end sequencing was performed using Illumina’s Solexa Genome Analyzer II using the standard protocol. The image deconvolution and calculation of quality value were performed using Goat module (Firecrest v.1.4.0 and Bustard v.1.4.0 programs) of Illumina pipeline v.1.4. Sequenced reads were generated by base calling using the Illumina standard pipeline.

Genome sequence and sRNA annotation information of *Synechocystis* were downloaded from NCBI and Mitschke et al. [3]. sRNA sequence reads were pre-processed using NGS QC Toolkit (Version: 2.3) to remove low-quality bases and adapter sequences. Reads after QC were aligned to the *Synechocystis* genome using Burrows-Wheeler Alignment tool software version 0.7.10 with perfect match parameters. Raw counts of reads that uniquely mapped to each sRNA region were calculated by HTseq (Version: 0.6.1). Then read counts were normalized to the aligned RPKM to obtain the relative expression levels.

### qRT-PCR analysis

The RNA extraction method was the same as described above. cDNAs were synthesized using SuperScript^®^ VILO™ cDNA Synthesis Kit following manufacturer’s protocol (Invitrogen, Carlsbad, CA) and 1 μL of each dilution was used as template for following qRT-PCR reaction. The qPCR reaction was carried out in 10 μL reactions containing 5 μL of UltraSYBR Mixture (CW Biotech, Beijing, China), 3 μL dH_2_O, 1 μL dilute template cDNA, and 1 μL of each PCR primer, employing the StepOnePlus™ Real-Time PCR System (Applied Biosystems, Foster City, CA) [36]. Three technical replicates were performed for each sample. Data analysis was carried out using the StepOnePlus analytical software (Applied Biosystems, Foster City, CA) and the 2^−ΔΔCT^ method [52]. 16S rRNA was selected as a reference gene, and the data were presented as ratios of the amount of normalized transcript in the treatment to that from the WT control.

### RT-PCR analysis

The RNA extraction method was the same as described above. cDNAs were synthesized using specific reverse primer of Ncl1460 named qRT-ncl1460-R (Additional file 2: Table S3) and SuperScript^®^ VILO™ cDNA Synthesis Kit following manufacturer’s protocol (Invitrogen, Carlsbad, CA). Then 1 μL cDNA was used as template for RT-PCR using primer qRT-ncl1460-F and qRT-ncl1460-R.

### Northern blotting

The non-radioactive northern blot method to detect small RNAs was performed following Kim et al. (2010)’s work [53]. Briefly, 10 μg total RNA was extracted from WT for blotting assays. Oligonucleotides probe of CoaR (5′-GTTAAGATATAGAGACAATTTAGCTGA-3′) was added digoxin-label using Roche DIG Oligonucleotide Tailing Kit II (F. Hoffmann-La Roche Ltd, Switzerland) for hybridization. Anti-digoxigenin-AP and CSPD (F. Hoffmann-La Roche Ltd, Switzerland) were used to detection.

### 3**′** Race

Total RNA of WT was extracted as described above. Then RNA was added with a poly(A) tail using NEB *E. coli* poly(A) polymerase (New England Biolabs Inc., MA, USA). After that a specific primer containing oligo dT (5′-CACACAGGAAACAGCTATGACCATGTTTTTTTTTTTTTTTTTT-3′) and SuperScript^®^ VILO™ cDNA Synthesis Kit were used to synthesize cDNA. At last, forward primer (5′-CCTTTCCCATAAGTCTAATTGCTTAGAGG-3′) and reverse primer (5′-CACACAGGAAACAGCTATGACCATG-3′) were used to amplify Ncl1460 containing 3′ end. PCR product was purified and ligated into pTZ57R/T using InsTAclone PCR Cloning Kit (Thermo Fisher scientific Inc., MA, USA) for sequencing analysis.

### qRT-PCR for adaptive laboratory evolutionary strains

In this study, seven evolved strains from previous studies [20], i.e., E1, E2, E3, E4, E5, E6, and E7 which could, respectively, endure 0.20, 0.25, 0.30, 0.35, 0.40, 0.45, and 0.50% (*v/v*) 1-butanol, were selected and cultured in normal BG11 medium along with WT and all the samples were harvested at 48 h for sRNA extraction and qRT-PCR analysis.

### Mutant strains construction

*Escherichia coli* DH5α strain was used for vectors construction and enrichment. Primers used in this study were listed in Additional file 2: Table S2. All the plasmids and mutants constructed in this study were concluded in Additional file 2: Table S6. For overexpression and suppression of sRNAs, fragments of sRNAs and related reverse complementary chain were amplified from the *Synechocystis* genome with specific primers that introduced *Xba*I and *BamH*I restriction sites and ligated to a broad host replicating vector pJA2 with anti-kanamycin cassette (kindly provided by Prof. Paul Hudson of KTH Royal Institute of Technology of Sweden) [10, 54]. *Synechocystis* was transformed by electroporation (~10 ng plasmid DNA) using GenePulser Xcell (Bio-rad, Hercules, CA) and grown photoautotrophically on agar plate adding 10 μg/mL kanamycin. The transformants were validated by colony PCR.

For GFP reporting assays in *Synechocystis*, genome sequence between *sll0821* and *slr0847* containing the promoter region of *slr0847* was amplified through PCR and fused to GFP sequence, then the fused fragment was introduced with *Kpn*I and *EcoR*I restriction site and ligated to pXT37b (digested with *Kpn*I and *EcoR*I restriction enzymes to remove the promoter region; this vector was kindly provided by Dr. Xuefeng Lu of Qingdao Institute of Bioenergy and Bioprocess Technology of Chinese Academy of Sciences) [55]. The constructed vector was introduced into the CoaR(+), CoaR(−) and WT strains through electro-transformation and grown photo-autotrophically on agar plate containing 10 μg/mL spectinomycin with or without 10 μg/mL kanamycin, respectively. The transformants were validated by colony PCR and purified through passages on plates with increased antibiotics.

### Growth patterns and fluorescence measurement

For measurements of growth patterns, 5 mL fresh cells at OD_630_ of 0.2 were collected by centrifugation at 3000×*g* and 4 °C and were then inoculated into 25 mL of BG11 liquid medium in a 100-mL flask; 1-butanol treatment was carried out by adding 0.20% (*v/v*) 1-butanol (Merck, USA) to the medium at beginning of the cultivation, each with three replicates and repeated at least three times. Cell density was measured on a UV-1750 spectrophotometer (Shimadzu, Japan) at OD_730_ or on an ELx808 Absorbance Microplate Reader (BioTek, Winooski, VT, USA) at OD_630_. Culture samples (1 mL or 200 μL, respectively) were taken and measured at both OD_730_ and OD_630_ every 12 h.

For GFP reporting assays, *Synechocystis* strains were harvested by centrifugation (4 °C, 7800×*g*) and re-suspended with ddH_2_O after 72 h. The final OD of the re-suspensions was measured on UV-1750 spectrophotometer and the GFP fluorescence was measured using an F-2700 fluorescence spectrophotometer (Hitachi, Japan) at EX 395 nm and EM 509 nm. The data were normalized by respective OD and the fluorescence of wild type *Synechocystis*, each with three replicates and repeated at least three times.

### 3′-Dephospho-CoA and CoA detection and quantitation

The standard dPCoA and CoA were purchased from Sigma-Aldrich (St Louis, USA). The mass spectra of two substances were determined using LC/MS. The standard curve of dPCoA and CoA was plotted through measuring the peak area of a series of dilutions of the respective standard, each dilution with 3 replicates using LC/MS (Additional file 1: Fig. S6). The extraction of total metabolites from *Synechocystis* and LC-MS analysis was conducted using the protocol described before [20]. A total of 36 samples, including WT, the CoaR(+) and CoaR(−) strains grown in normal BG11 medium or BG11 medium with 0.25% (*v/v*) 1-butanol were harvested at 60 h, each with six biological replicates. The quantitation of dPCoA and CoA of each sample were measured through standard curve, respectively.

### Cell size measurement using flow cytometry

Flow cytometric analysis was performed on a Calibur fluorescence-activated cell sorting (FACS) cytometer (Becton Dickinson) to reveal cell size differences among WT, CoaR(+) and CoaR(−) strains with the similar settings described previously [13]. Cells were harvested at 48 h and data analysis was conducted using the CellQuest software, version 3.1 (Becton Dickinson). The results were repeated by at least three times.

### Lipid profile measurement

Total lipids from approximate 5-mg dry cells of the WT, CoaR(+), and CoaR(−) strains (each with 3 biological replicates) were extracted using 2 mL chloroform/methanol (*v*/*v*, 2/1) ultrasonic treatment for 10 min and centrifugation at 4000 rpm for 5 min. The collected supernatants were dried under nitrogen flow and then at 60 °C until the weight of samples remained constant. The samples were weighed and moved to 10-mL flasks. Then, 5 mL 2.0% H_2_SO_4_–methanol (*v*/*v*, H_2_SO_4_/methanol) was added, and the flask was stirred at 70 °C for 1 h. Then 2 mL of hexane and 0.75 mL of distilled water were added to the flask and mixed and the upper hexane layer contained the fatty acid methyl esters (FAMEs). The hexane layer was transferred to a new vial and mixed with the internal standard C17-ME for analysis by gas chromatography (GC). FAME analyses were carried out by an Agilent 6890 GC instrument and FAME yield was calculated using the equation described by Liu et al. [32].

### Quantitative proteomics and data analysis

Samples composed of the WT and the mutant CoaR(+) were harvested after cultivation for 48 h under 0.20% (*v/v*) 1-butanol stress, each with two replicates for quantitative isobaric tags for relative and absolute quantification (iTRAQ) liquid chromatography-tandem mass spectrometry (LC-MS/MS) proteomic analysis. Protein preparation and digestion, iTRAQ labeling, LC-MS/MS proteomic analysis, and proteomic data analysis were the same as before [36]. Briefly, cell samples for proteomics analysis were collected by centrifugation at 8000×*g* for 10 min at 4 °C and immediately frozen in liquid nitrogen. After proteins preparation, the iTRAQ labeling of peptide samples was performed using iTRAQ Reagent 8-plex Kit (Applied Biosystems, Foster City, CA) according to the manufacturer’s protocol. Then the mass spectroscopy analysis was performed using an AB SCIEX TripleTOF™ 5600 mass spectrometer (AB SCIEX, Framingham, MA, USA), coupled with online micro flow HPLC system (Shimadzu, JAPAN). The MS data were processed using Proteome Discoverer software (Version 1.2.0.208) (Thermo Scientific) with default parameters to generating peak list. Genome sequence and annotation information of *Synechocystis* were downloaded from NCBI and the Comprehensive Microbial Resource (CMR) of TIGR (http://www.tigr.org/CMR) [56]. Proteins with 1.2-fold change between the CoaR(+) strain and control WT samples and *p*-value of statistical evaluation <0.05 were determined as differentially expressed proteins.

## Additional files

**Additional file 1: Fig. S1.** Mutant constructions using different vectors. **A)** Overexpression of sRNAs using pJA2. **B)** Suppression of sRNAs using pJA2. **C**) Gene overexpression using pXT37b. **Fig. S2 A)** Growth phenotypes of WT, Ncl1460(+), and Ncl1460(−) under BG11 media with 4.6 μM CdSO_4_ **; B)** Growth phenotypes of WT, Ncl1460(+), and Ncl1460(−) under BG11 media under pH6.1; **C)** Growth phenotypes of WT, Ncl1460(+), and Ncl1460(−) under BG11 media with 4% (*w/v*) NaCl. **Fig. S3 A)** Northern blotting detection of Ncl1460. **B)** 3’ RACE results of Ncl1460. **C)** RT-PCR results of Ncl1460 in WT, Ncl1460(+), and Ncl1460(−). **Fig. S4** Predicted functional regions of Ncl1460 using CopraRNA. **Fig. S5** Predicted interaction regions of Ncl1460 on its target genes using CopraRNA. **Fig. S6** Standard curves for measurements of CoA **A)** and dPCoA **B)**. **Fig. S7** Description of the proteomics data. **A)** Basic information statistics including the total spectrum, unique spectrum, peptides, unique peptides, and identified proteins. **B)** Peptide length distribution. **C)** Distribution of protein’s sequences coverage. The different colors represent different coverages, and the number in each color represents the identified proteins.

**Additional file 2: Table S1**. Mapping results and the expression profiles of all identified sRNAs. C represents Synechocystis cultured in normal BG11 while B represents medium with butanol. The RPKM value was reads per kilobase per million mapped reads. The fold change of each ncRNAs was shown as B/C. **Table S2** sRNAs with regulated expression at 24 h, 48 h, and 72 h. **Table S3** Primers used in this study. **Table S4** Constructed sRNA mutants in this study. **Table S5** Regulated proteins by CoaR. **Table S6** plasmids and strains constructed in this study.

## Abbreviations

dPCoA: 3′-dephospho-CoA
LC/MS: liquid chromatography–mass spectrometry
qRT-PCR: quantitative real time polymerase chain reaction
RT-PCR: reverse transcriptional polymerase chain reaction
3’ RACE: 3′ rapid amplification of cDNA end
RPKM: reads per kilobase per million mapped reads
sRNA: small RNA
sRNome: small RNA transcriptome
