# Supplementary figures and images for: A novel small RNA CoaR regulates coenzyme A biosynthesis and tolerance of Synechocystis sp. PCC6803 to 1-butanol possibly via promoter-directed transcriptional silencing
Source: Biotechnol Biofuels. 2017 Feb 20;10:42. doi: 10.1186/s13068-017-0727-y (PMC5319066; doi:10.1186/s13068-017-0727-y)

## Slide 1
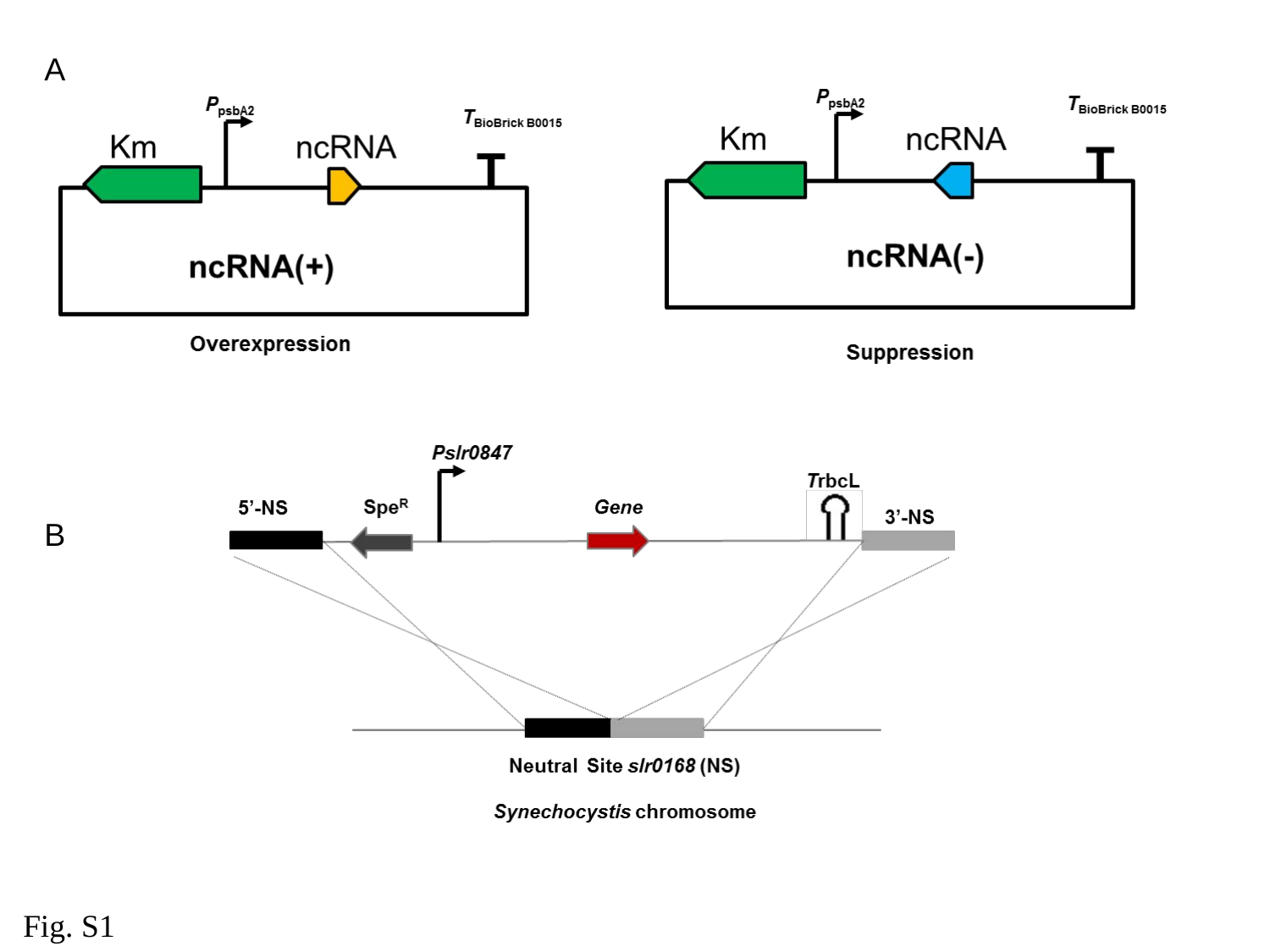

A
B
Fig. S1

## Slide 2
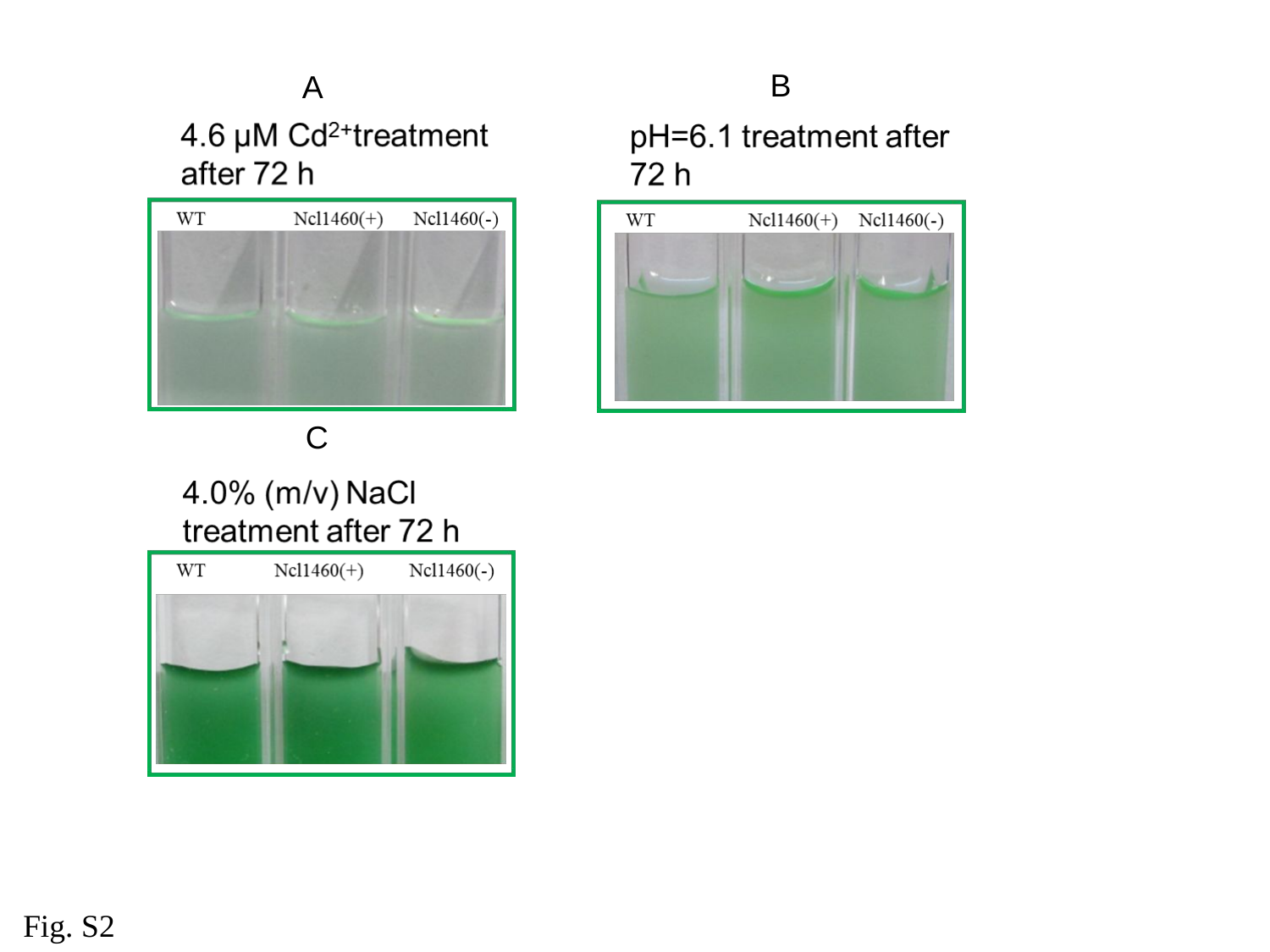

B
A
C
Fig. S2

## Slide 3
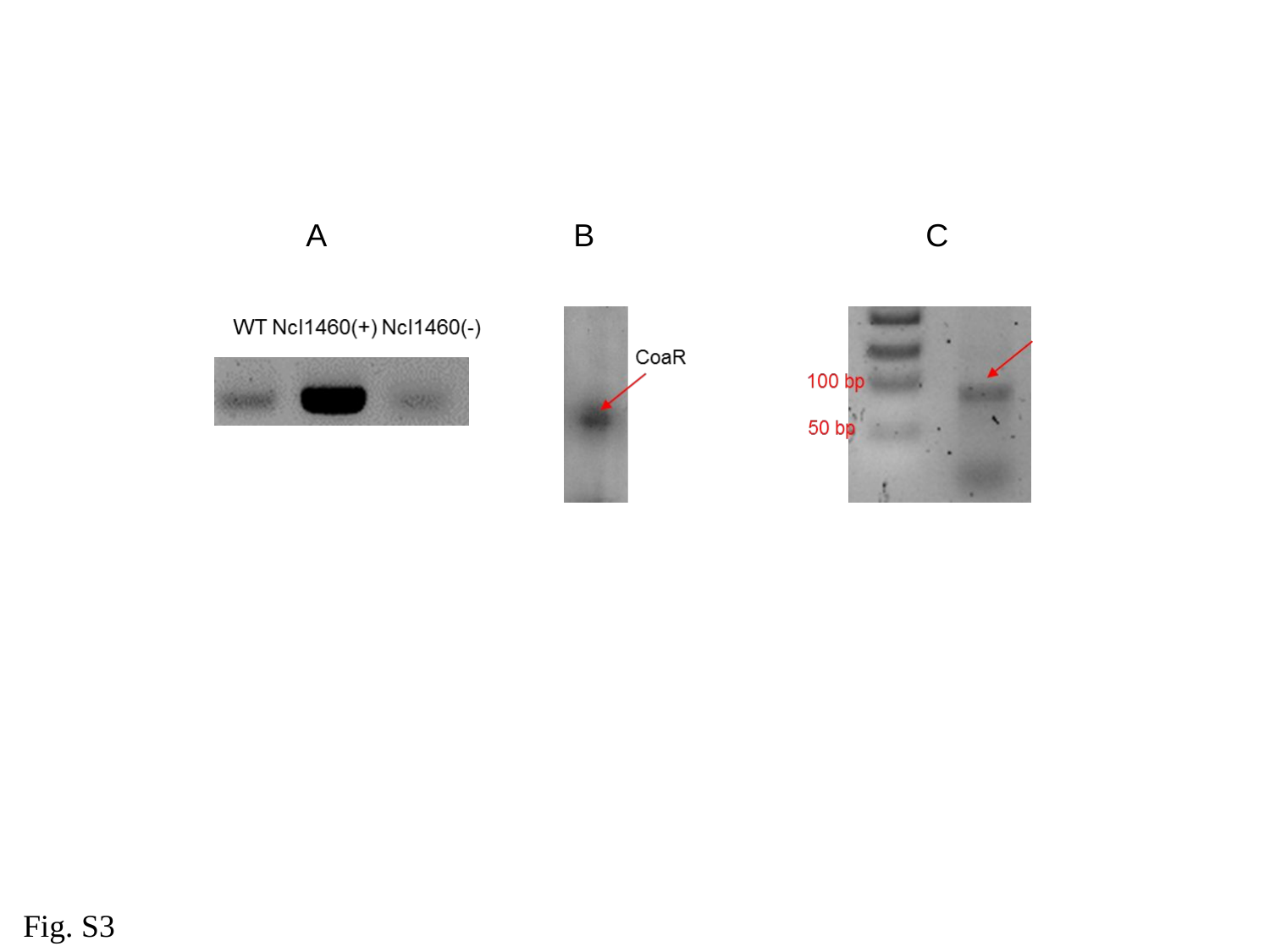

A
B
C
Fig. S3

## Slide 4
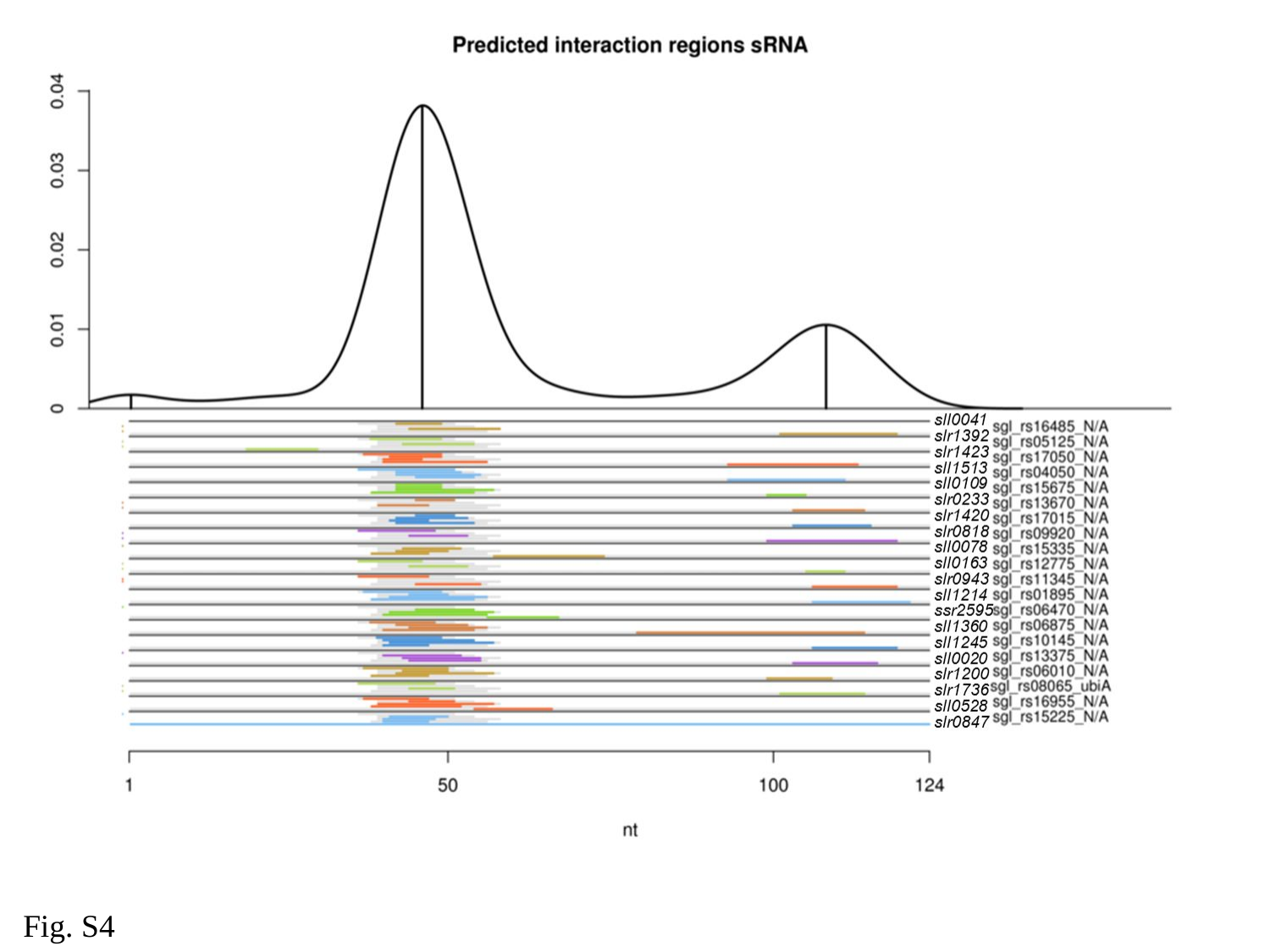

Fig. S4

## Slide 5
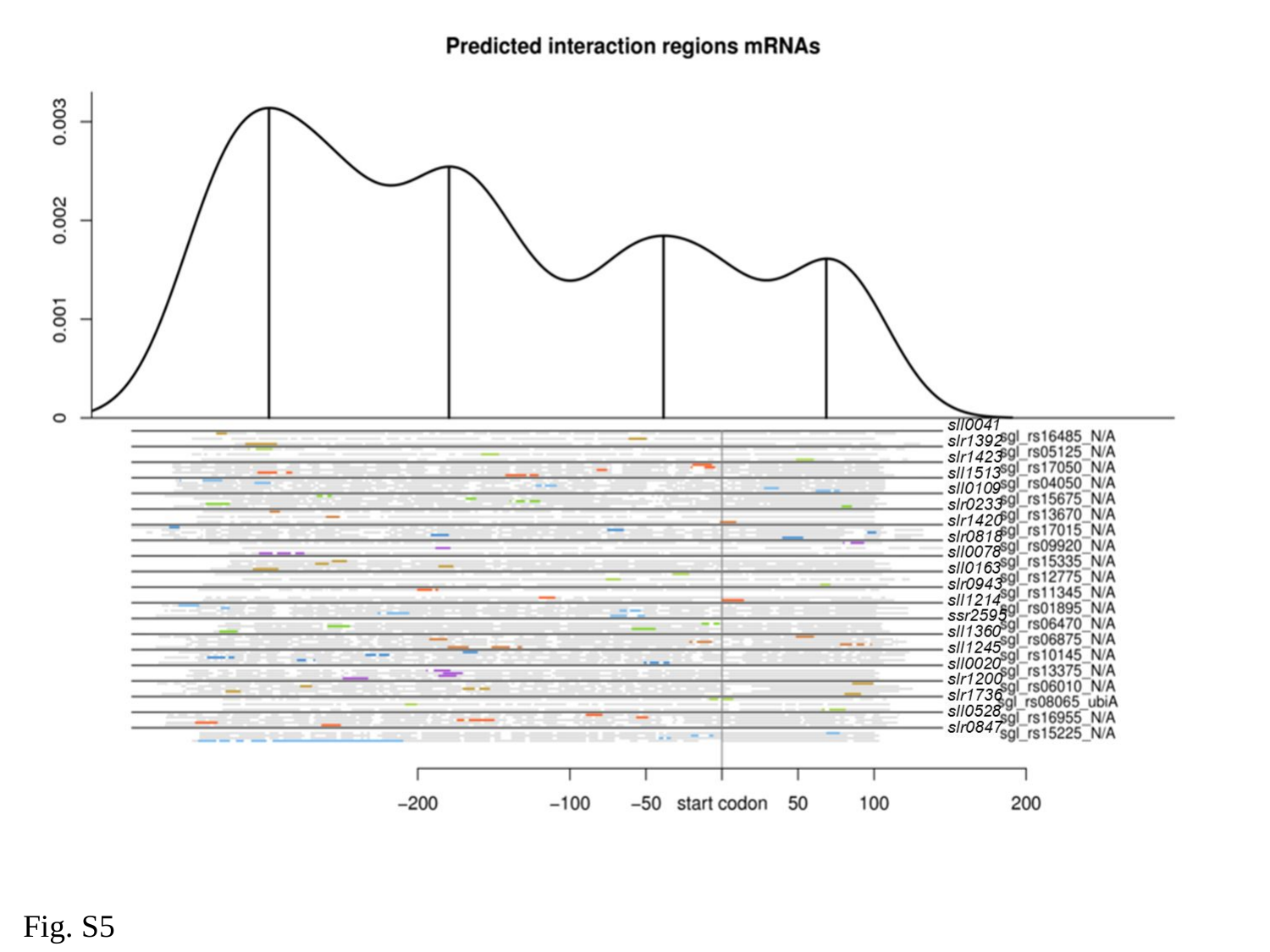

Fig. S5

## Slide 6
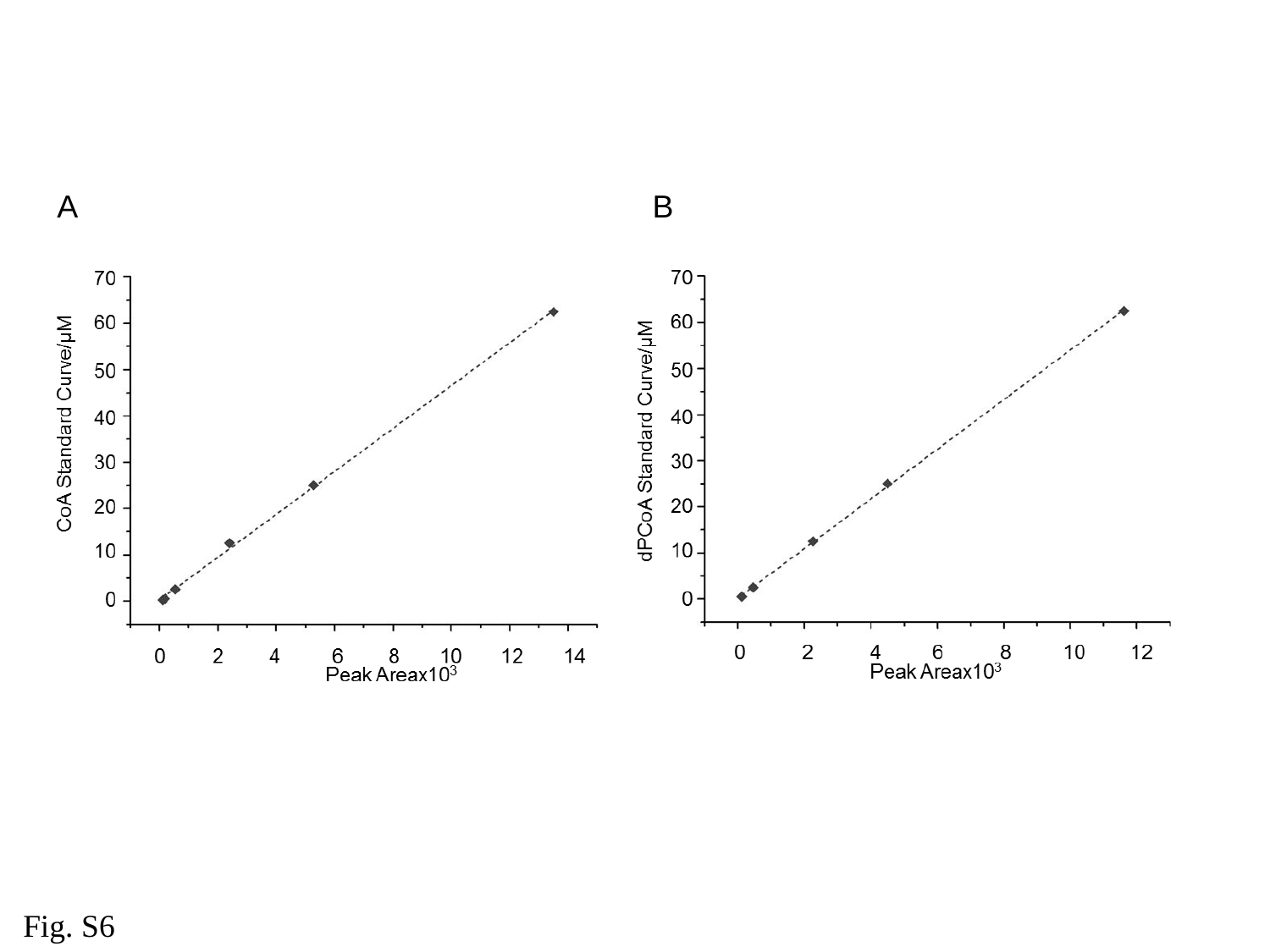

A
B
Fig. S6

## Slide 7
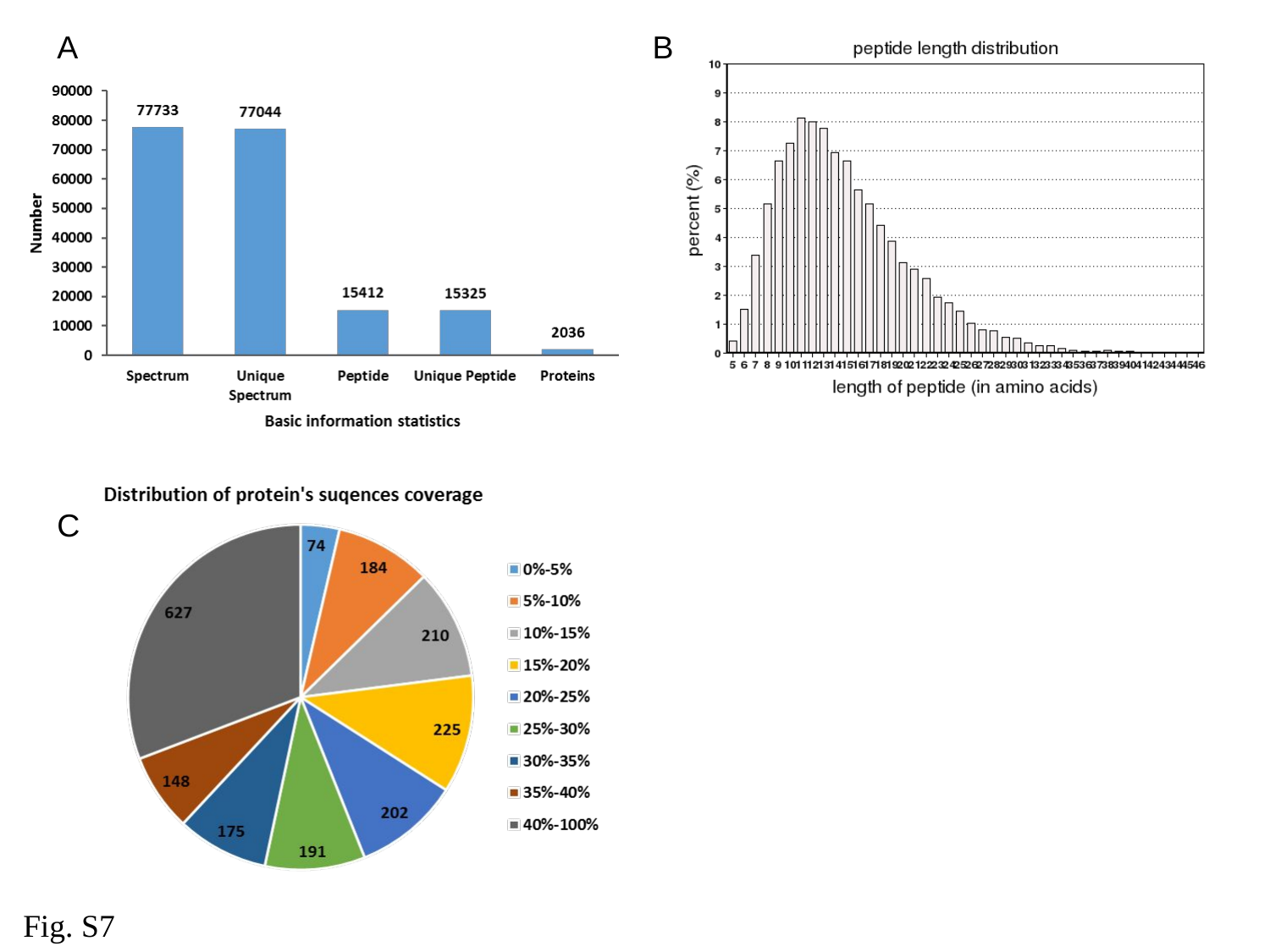

A
B
C
Fig. S7

Supplement: Supplementary file 1 — Additional file 1: Fig. S1. Mutant constructions using different vectors. A) Overexpression of sRNAs using pJA2. B) Suppression of sRNAs using pJA2. C) Gene overexpression using pXT37b. Fig. S2 A) Growth phenotypes of WT, Ncl1460(+), and Ncl1460(−) under BG11 media with 4.6 μM CdSO4 ; B) Growth phenotypes of WT, Ncl1460(+), and Ncl1460(−) under BG11 media under pH6.1; C) Growth phenotypes of WT, Ncl1460(+), and Ncl1460(−) under BG11 media with 4% (w/v) NaCl. Fig. S3 A) Northern blotting detection of Ncl1460. B) 3’ RACE results of Ncl1460. C) RT-PCR results of Ncl1460 in WT, Ncl1460(+), and Ncl1460(−). Fig. S4 Predicted functional regions of Ncl1460 using CopraRNA. Fig. S5 Predicted interaction regions of Ncl1460 on its target genes using CopraRNA. Fig. S6 Standard curves for measurements of CoA A) and dPCoA B). Fig. S7 Description of the proteomics data. A) Basic information statistics including the total spectrum, unique spectrum, peptides, unique peptides, and identified proteins. B) Peptide length distribution. C) Distribution of protein’s sequences coverage. The different colors represent different coverages, and the number in each color represents the identified proteins. [file 13068_2017_727_MOESM1_ESM.ppt]
